# Red List of vascular plants of Tajikistan – the core area of the Mountains of Central Asia global biodiversity hotspot

**DOI:** 10.1038/s41598-020-63333-9

**Published:** 2020-04-10

**Authors:** Arkadiusz Nowak, Sebastian Świerszcz, Sylwia Nowak, Hikmat Hisorev, Ewelina Klichowska, Anna Wróbel, Agnieszka Nobis, Marcin Nobis

**Affiliations:** 1 Institute of Biology, University of Opole, Opole, 45-052 Poland; 2 Botanical Garden, Center for Biological Diversity Conservation, Polish Academy of Sciences, Warszawa, 02-976 Poland; 3 Institute of Botany, Plant Physiology and Genetics, Tajik Academy of Sciences, Dushanbe, 734017 Tajikistan; 4 Faculty of Biology, Jagiellonian University, Kraków, 30-387 Poland; 5 Research laboratory ‘Herbarium’, National Research Tomsk State University, Tomsk, 634050 Russia

**Keywords:** Ecology, Conservation biology

## Abstract

Central Pamir-Alai, which is located almost entirely within the area of Tajikistan, is one of the world hotspots of biodiversity, harbouring ca. 4,300 species and 1,400 endemic plants. The first application of the IUCN Red List criteria reveals that among all native species occurring in Tajikistan 1,627 taxa (38.11%) are threatened, including 23 extinct (0.54%), 271 (6.34%) critically endangered (CR), 717 (16.79%) endangered (EN) and 639 (14.96%) vulnerable (VU). Globally, 20 taxa are extinct, 711 (16.65%) threatened, including 144 (3.37%) critically endangered, 322 (7.54%) endangered and 245 (5.73%) vulnerable. As we found positive correlation between human density and the number of threatened species, we suspect this indirect factor responsible for the species diversity decline. Extinct or threatened taxa have short blooming periods in spring or early summer, have limited geographical range and inhabit mainly valley bottoms at lower altitudes. Threatened taxa occupy extremely dry or wet habitats, such as deserts, semi-deserts, water reservoirs and fens. The group of threatened plants consists mostly of Central Asian, Indo-Indochinese and Arctic species. Ornamental plants have a higher extinction risk than other plants, but species collected for medicinal reasons and used for forage or food reveal lower retreatment rate. Our assessment fills a gap for important plant area and provides the data for raising the effectiveness of plant diversity conservation.

## Introduction

Species diversity loss still remains one of the main imperatives of our time and therefore one of the main topics of scientific studies. Currently, hundreds of plant species and many habitat types are globally threatened^1,2^. A range of factors are responsible for these declines, with human population growth, habitat fragmentation and climate change regarded as the most crucial^3^. The continuing decline of plant diversity demands continuous research on evaluation of the conservation status of flora with the use of comprehensive International Union for Conservation of Nature (IUCN) criteria (www.iucnredlist.org). These criteria are widely recognised as the most comprehensive tool for assessing the global conservation status of species and categorising plants according to their estimated risk of extinction (e.g. Orsenigo *et al*.^4^, Maes *et al*.^5^). As the most effective conservation actions, policies and law implementation take place at the national scale, numerous countries have established national lists of threatened species with the use of IUCN criteria and guidelines at regional levels (Rossi *et al*.^6^). Despite some biases and shortcomings of scientific foundations^7^, red lists are widely accepted as an appropriate measure for setting conservation priorities. In some countries, including Tajikistan, they also have a legal status and directly influence the state governance of the plant diversity^8^. However, based on the information available in the National Red List Database^9^, almost 20% of Eurasian countries still have no available red lists for vascular plants, as well as studies on threatened species being generally underrepresented^10^.

The territory of Tajikistan, with its phytogeographical complexity, is extremely diverse in terms of climate, landscape and habitat conditions. This has resulted in speciation of many altitudinal and ecological vicariants that occur in many cases in single, isolated valleys or mountain ridges (e.g. within *Scutellaria* or *Asperula* genera). According to the ten-volume study of the flora of the former Soviet Tajik Republic^11–20^ supplemented by the works of Zakirov^21^, Tzvelev^22^, Ikonnikov^23^ and more recently by, for example, Nobis *et al*.^24,25^ and Nowak & Nobis^26^, the vascular flora of the Pamir-Alai consists of ca. 4,300 species assigned to 116 families. Approximately 30% of vascular plants species known from Tajikistan are generally accepted as endemics^27^.

Because of its floristic richness, the territory of Tajikistan is recognised by Conservation International as a hotspot of biodiversity^28^ and, simultaneously, is regarded as the most sensitive to climate change and biodiversity loss^29^. But still only twelve species from this country are listed as globally threatened (e.g. Darvaz dogwood *Swida darvasica* and wild apple *Malus sieversii*). Additionally, Middle Asian mountainous temperate forests and steppes are regarded as a vulnerable ecoregion of the world^30^.

Despite the extraordinary floristic richness, there is still no credible and valid information as to how many vascular plant species, especially endemics, are threatened in Tajikistan, which regions of the country are the richest in endangered plants, and which factors affect this threat the most. The red books of the country, consecutively issued in 1988 and 2015, include first 209 and then 239 threatened vascular plants^31,32^. These numbers are surprisingly low, given that the threats from urbanisation, agriculture and climate change in Tajikistan are extremely high.

The specific aims of our research was to (1) compile the preliminary checklist of vascular plant species of Tajikistan from all available sources, (2) to evaluate the threat status of all native plant taxa in Tajikistan using the IUCN guidelines and criteria, (3) to highlight the threat status of Tajiks endemics at a global level, (4) to find out the relation of a particular endangered group to biological, ecological and climatic factors such as habitat, altitude, life form, blooming season, endemism status and flower colour, and (5) to identify the subregional hotspots of threatened flora in the territory of the country. Our study is a comprehensive completion of the former approaches, providing the first assessment of the whole flora for the country in the Mountains of Central Asia biodiversity hotspot.

## Material and methods

### Study area

Tajikistan covers 143,500 km^2^ and is a typically mountainous country, with more than 50% of the area located above 3,000 m a.s.l. According to the bioclimatic classification, the study area can be classified within the Mediterranean type of macrobioclimate^33^. However, recent research on the southwestern and Central Asian bioclimate suggests that the Irano-Turanian bioclimatic zone should be distinguished by higher continentalism, lower precipitation, a longer dry season and lower winter temperature minima^34^. The area has a generally high level of solar insolation (2,090–3,160 hours of sunshine), as well as a low percentage of cloud cover, high-amplitude annual temperatures and moderate humidity and precipitation, with the exception of the spring period when there is a considerable amount of rainfall. In the alpine belt of high mountains the climate is much harsher, with the average temperatures in July between 9.7° and 13.5 °C. Annual precipitation ranges in the western Pamir-Alai from ca. 350 mm (Zeravshan Mts.) to ca. 600 mm in the Hissar Range (in some locations up to 2,000 mm). In the western part of the country, the lower limit of permanent snow is at an altitude of 3,500–3,600 m a.s.l.; in its eastern regions, at 5,800 m a.s.l.^35–37^.

### Species checklist and red list data

As there is no available checklist of vascular plants, the first stage of the study was to compile the checklist of all vascular plants that have ever been reported from the territory of Tajikistan. The main source of information was the ten-volume flora of the country with several important supplementations^11–23^. This produced 138,203 records coming from the research between 1880–1985. Additionally, the authors used the data from the phytosociological research stored in the Vegetation of Middle Asia data set, with 26,357 records from 2006–2019^38,39^. We also used data from our herbarium collections gathered during 34 field surveys (2006–2019) stored in KRA (Jagiellonian University) and OPUN (Opole University) as well as 40 herbaria, with the four most important (TAD, LE, TASH and KHOR; herbarium codes after Thieris^40^) being visited personally by the authors or asked for specimen loans. We include in our checklist species with reliable distributional data. Lower level taxa (varieties and forms) as well as hybrid taxa were excluded to avoid misinterpretation. Similarly, some alien species reported by Nowak *et al*.^41^ as recent newcomers to the country were also excluded from the analyses.

As only herbarium stations and phytosociological records were georeferenced, we used the geobotanical division at subregional level (26 units; Fig. S1a) to visualise the distribution of the taxa^42^. The assessments were mostly based on criterion B1 (extent of occurrence), however when consistent data on population size, number of locations or trends were available, other criteria were also applied (i.e. A, B2, C, and D^1^). The extent of occurrence was defined using the minimum convex polygon method which determines the area contained within the shortest continuous imaginary boundary that can be drawn to encompass all the subregions occupied by a taxon. The glaciers and ice fields were excluded from the count. The area of occupancy (criterion B2) was defined as the area within the Extent of Occurrence (EOO) currently occupied by a given taxon. To apply sub-criteria under the criterion B, distribution data have been used to define the number of locations and the occurrence of severe fragmentation for each taxon according to IUCN^1^ guidelines. Following IUCN guidelines, for the endemic plants of Tajikistan we consider the national threat status as global one – showing the importance of our research for a global evaluation of the plants’ endangerment.

The severe fragmentation has been evaluated by estimating the fraction of the taxon occurring in isolated populations. A distance of 50 km was set as a general isolation threshold^4^. For estimating continuing decline, historical habitat or population trends were considered when available; in the absence of such data, the evaluation was founded on expert judgment. A taxon was considered extinct (EX) if it was not recorded during the last 50 years, when recent field surveys focused on finding the species in its historical area of occurrence were unsuccessful and, simultaneously, that its typical habitat(s) was/were assessed as the most threatened in Tajikistan (riverside forests, alpine forests, broad-leaved forests, meadows and pastures, xerothermophilous shrubs, riverbed shrubs and xerothermophilous dwarf bushes^27^).

The data on altitudinal and geographical distribution of the species as well as life form (according to Raunkier), phenology, flower colour, habitat preferences and usefulness were extracted from the ten-volume flora of Tajikistan and field surveys of the authors (particularly habitat requirements, Table S1). Based on the collected data, we have prepared diagrams showing the proportions of species of different threat categories in relation to all the variables. In the graph with the elevation gradients referred to as CR, EN and VU threat categories, we used normalisation of data using z-scores expressed as standard deviations from the average for a given threat category. In addition, Kruskall–Wallis test and a posteriori Dunn’s test were performed to check the differences in flowering duration between the threat categories. The correlation between the number of species with the threat category CR, EN, VU, RE, EX and the median population density of each geobotanical subregion in Tajikistan was calculated using Pearson’s correlation. Visualisations and tests were prepared in R version 3.5.1^43^. Phytogeographical division and elements were applied after Grubov^44^ and Takhtajan^45^ supplemented by Nowak *et al*.^41^. Species nomenclature mainly followed Cherepanov^46^.

## Results

The list of Tajik vascular plants comprises 4,269 species and subspecies (Table S1). Thirty-two taxa are recognised as extinct in the country, including 20 endemics which, therefore, are categorised as extinct globally, e.g. *Allium pauli*, *Bellevalia atriviolacea*, *Crataegus darwasica*, *Echinops pseudomaracandicus*, *Gagea minutissima*, *Hedysarum cisdarvasicum*, *Juno tadshikorum*, *Onobrychis gontscharovii*, *Ranunculus mogoltavicus* and *Tulipa anisophylla*. These plants were known only from a single location (with the exception of *Crategus darwasica*), mainly in xerothermophilous shrubs, alpine swards and riverside forests and bushes.

**Figure 1:**
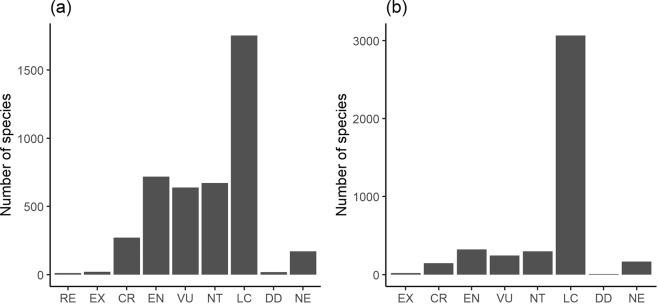
Number of species of threat categories for the flora of Tajikistan at (**a**) national and (**b**) global level.

At a national level, 1,627 taxa (38.11%) have been assessed as threatened, including 271 (6.34%) critically endangered (CR), 717 (16.79%) endangered (EN) and 639 (14.96%) vulnerable (VU) (Fig. 1a). Globally, 711 taxa (16.65%) have been classified as threatened, including 144 (3.37%) critically endangered, 322 (7.54%) endangered and 245 (5.73%) vulnerable (Fig. 1b).

Considering the near threatened (NT) species, 671 taxa (15.71%) have been assigned to this category at a national level, and 296 plants (6.93%) at a global one. Overall, the highest percentage of the taxa have been assessed as not demanding current conservation and classified as least concern (LC): 1,754 (41.08%) at a national level, and 3,067 taxa (71.84%) at a global one.

Seventeen taxa have been assigned to the data deficient (DD) category as no credible records were found or their taxonomic status was questionable. A group of 169 species was identified as alien species to the flora of Tajikistan and not evaluated (NE).

The species richness is not evenly distributed across geobotanical subregions in Tajikistan (Fig. S1b). The highest number of species is observed in Zeravshanian B (1,499 taxa), Hissaro-Darvasian A (1,440), South-Tajikistanian B (1,407) and South-Tajikistanian A (1,324)^41^.

The number of endangered and extinct species increases along with the increase in population density in each geobotanical subregion (Fig. S6).

**Figure 2:**
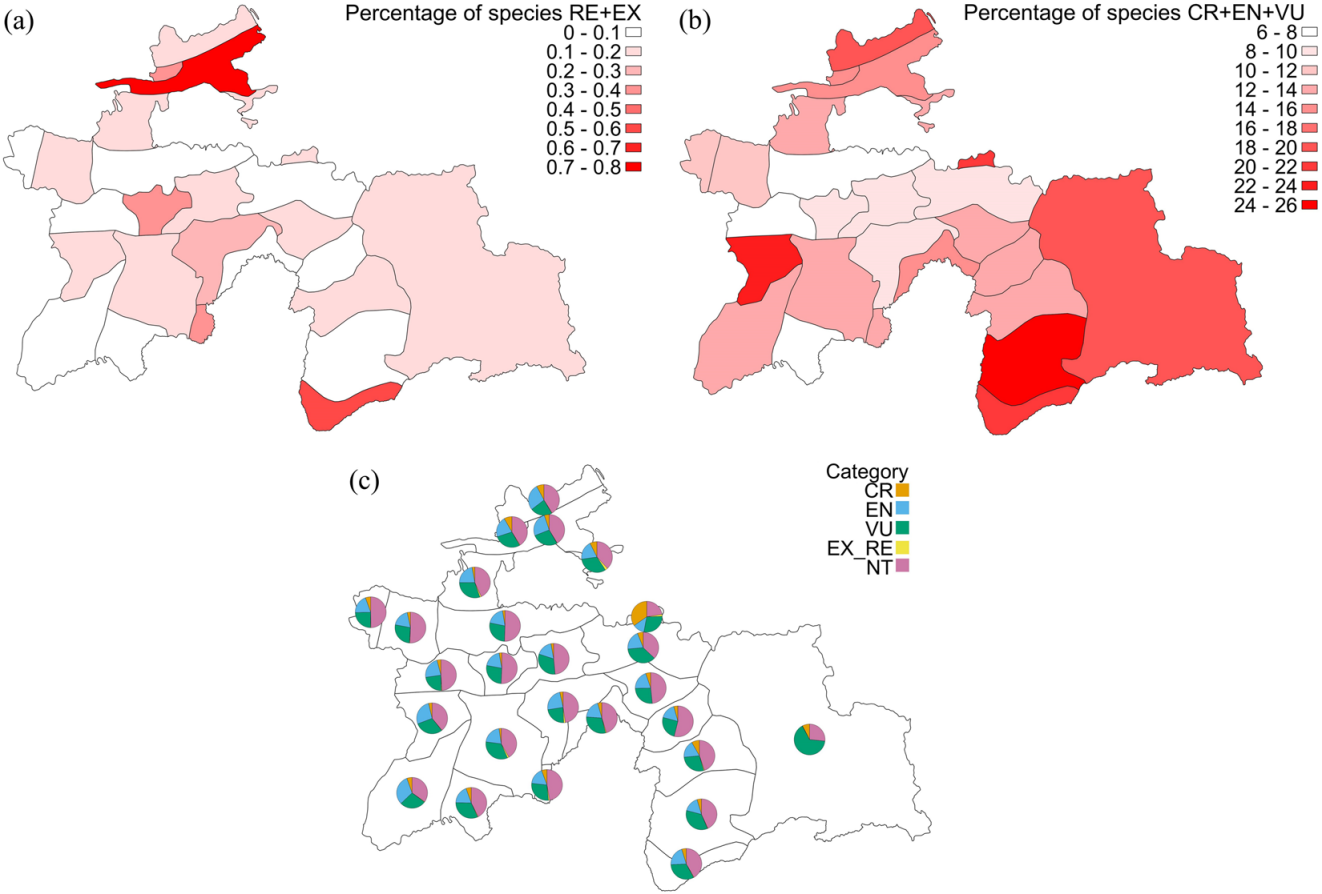
Percentage of species with (**a**) RE and EX, (**b**) CR, EN and VU threat categories and (**c**) percentage of threatened species (pie charts) in 26 phytogeographical subregions. Note that the species with RE + EX threat category on map (c) are not visible due to its negligible share.

The majority of the extinct (RE + EX) species were linked with the densely populated Syr Darya River Valley (Prisyrdarian subregion) and the Pyandzh River Valley (West Pamirian subregion; Fig. 2a). In both cases this group of taxa did not exceed 1% of the total species’ number of each subregion.

The number of threatened species (CR + EN + VU categories) varies across the country, ranging from 37 to 280 taxa per subregion (Fig. 2b). The highest number of such taxa was noted in the species-rich South Tajikistanian B subregion, an area strongly influenced by agriculture. The highest proportion of the threatened flora was found in the West Pamirian B (26%), South Tajikistanian A (22%) and Alaian (20%) subregions (Fig. 2b).

The highest proportion of the near threatened (NT) taxa was found in the East Tajikistanian C and Hissaro-Darwasian B subregions and the entire Zeravshan region, accounting for more than 50% of the total species’ number of each subregion (Fig. 2c).

With regard to the vertical distribution, the highest number of threatened species (CR + EN) was indicated for lowlands and colline zone (Fig. S2). There was an interesting exception for vulnerable (VU) taxa, with the number of threatened species lower than average between 1,400–3,000 m a.s.l., and above 3,000 m a.s.l. (Fig. S2).

**Figure 3:**
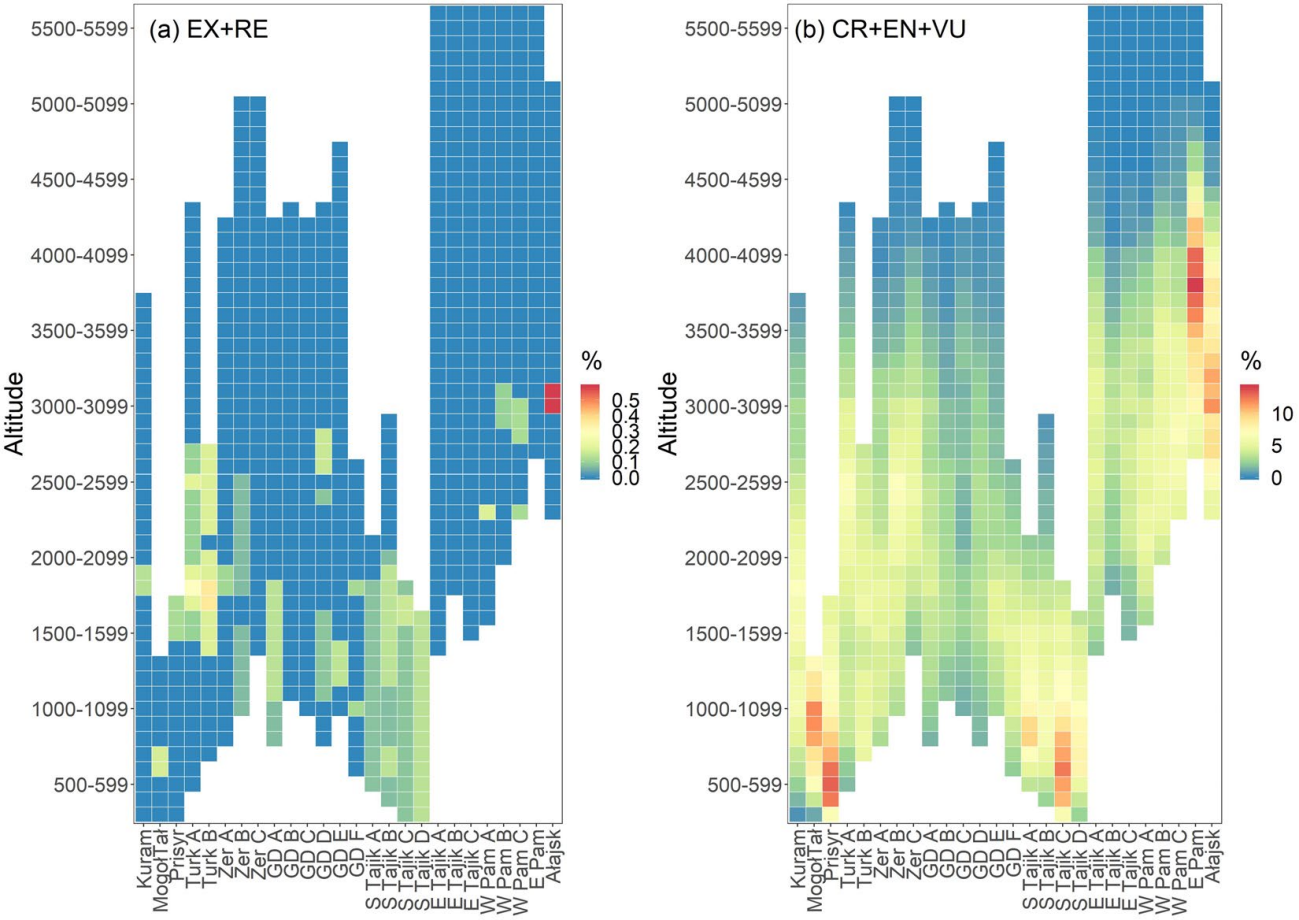
Percentage of the (**a**) extinct (RE, EX) and (**b**) threatened taxa (CR, EN, VU) in phytogeographical subregions (see Fig. S1a) with regard to its vertical span. Abbreviations: Kuram – Kuraminian, MogolTal – Mogoltausian, Prisyr – Prisyrdarian, Turk_A – Turkestanian A, Turk_B – Turkestanian B, Zer_A – Zeravshanian A, Zer_B – Zeravshanian B, Zer_C – Zeravshanian C, GD_A – Hissar-Darvasian A, GD_B – Hissar-Darvasian B, GD_C – Hissar-Darvasian C, GD_D – Hissar-Darvasian D, GD_E – Hissar-Darvasian E, GD_F – Hissar-Darvasian F, S_Tajik_A – South Tajikistan A, S_Tajik_B – South Tajikistan B, S_Tajik_C – South Tajikistan C, S_Tajik_D – South Tajikistan D, E_Tajik_A – East Tajikistan A, E_Tajik_B – East Tajikistan B, E_Tajik_C – East Tajikistan C, W_Pam_A – West Pamirian A, W_Pam_B – West Pamirian B, W_Pam_C – West Pamirian C, E_Pam – East Pamiraian, Alajsk – Alaian.

The altitudinal distribution of the red-listed taxa depends on the elevational range of a particular subregion. The biggest proportion of threatened (CR + EN + VU) taxa was noted for lowlands and colline belts in Southern Tajikistan C, Prisyrdarian, Mogoltavian as well as for the high altitude areas in Alai and plateaus of Eastern Pamir (Fig. 3a,b).

**Figure 4:**
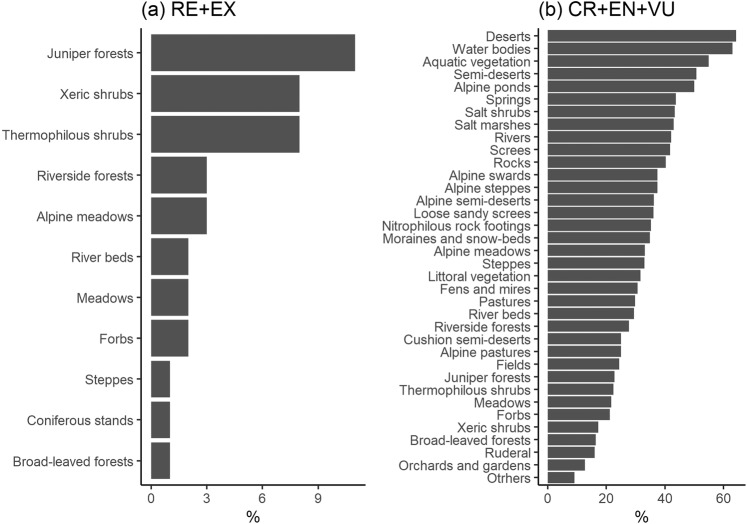
Percentage of the (**a**) extinct (RE, EX) and (**b**) threatened taxa (CR, EN, VU) in particular habitat type.

Generally, threatened (CR + EN + VU) taxa are linked with extremely dry or wet habitats, such as deserts and semi-deserts, water bodies, mires and fens, where they can reach up to 65% of the total number of species. On the other hand, the lower proportions of threatened taxa are noted in anthropogenic habitats (orchards, ruderal places), juniper forests, dry meadows and xerothermophilous shrubs (Fig. 4b).

Extinct species (RE + EX) in Tajikistan are related mostly to juniper forests as well as xerothermophilous and xeric shrubs (Fig. 4a).

The extinction risk of the species does not depend to a large extent on their growth form. However, there is a clear prevalence of nanophanerophytes, chamaephytes and cryptophytes in the threatened (CR + EN + VU) category and megaphanerophytes in the extinct (EX) group (Fig. S3).

**Figure 5:**
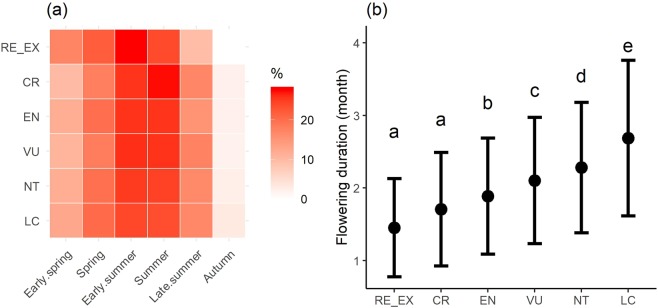
(**a**) Percentage of species with different threat categories with regard to their blooming season and (**b**) the duration of flowering period for the particular threat group (RE + EX, CR, EN, VU, NT, LC; Kruskal–Wallis test, chi-squared = 560.65, df = 5, *P* < 0.001). Different letters indicate significant differences among the threat groups and error bars represent standard deviation.

The critically endangered (CR) and endangered (EN) species bloom mainly in June, while the flowering peak of the extinct (RE + EX) plants was noted in May. The least concern (LC) and near threatened (NT) species are typically summer plants (Fig. 5a). More threatened and extinct taxa have a shorter flowering period than the non-threatened plants (Fig. 5b).

The extinct (RE + EX) species belong mainly to Pluriregional and Irano-Turanian phytogeographical elements. The group of threatened plants (CR + EN + VU) consists mostly of Central Asiatic (EI-T), Indo-Indochinese and Arctic taxa. The least concern (LC) species have a core distribution area in the Himalayan subprovince and Mediterranean province (Fig. S4).

An analysis of usage types in relation to threat categories indicates that ornamental plants (with beautiful large flowers and attractive colours) have a higher extinction risk than other plants. Similarly, this group was the most frequent in already extinct taxa. Plants collected for medicinal reasons, as well as species used for forage and food, reveal a lower threat level (Fig. S5a). The difference between the threat level of exploited and non-exploited species is lowest for the least concern taxa. Overall, the higher the threat level, the lower the proportion of the exploited plants (Fig. S5b).

## Discussion

### Significance of plant diversity threat assessment and conservation in Tajikistan

After the exclusion of anthropophytes, in total 4,160 species and subspecies have been assessed, which is a sample of around 50% of native species of Middle Asian flora (including Uzbekistan, Kyrgyzstan and Kazakhstan). This figure includes 1,273 exclusive endemics of Tajikistan, from which 20 are globally extinct and 144 critically endangered. As only twelve species from Tajikistan are included in the IUCN global red listing (6 CR, 3 EN and 3 VU, additionally 2 NT, 18 DD^47^), these numbers indicate the degree of under investigation of the conservation status in this valuable area of the world. This difference is particularly striking if we compare this region to other countries located in other biodiversity hotspots. In Italy, for example, 108 species were assessed to the CR, EN and VU categories, in India – 398 species, and in Indonesia – 481 species^1^. The Tajik’s natural heritage is under severe threat from climate change, habitat fragmentation and degradation caused by intensive grazing, population growth and unsustainable use of natural resources. All these phenomena and human activities have to be reflected in the threat status of the country flora, raising the numbers of endangered and vanished species in Tajikistan.

### Human population density – a prime suspect responsible for the loss of plant diversity?

The human population of the country is still increasing; since the middle of the last century this has been about six-fold^48^ and this can have a serious impact on the biodiversity^49^. Simultaneously the pastoral economy and the number of livestock has increased significantly^50^. Currently, in Tajikistan, 160,000 people depend on livestock production, whereas the potential carrying capacity of the area is between 3 and 5 times lower^51^. Livestock farming and grazing is highlighted as a major threat to vascular plants in many countries, particularly those with a pastoral culture (e.g. Bilz *et al*.^52^). One of the highly fragile subregions that face extinction (EX, RE categories) are the two river valleys of Syr-Daria (Prisyrdarian) and upper Pyandzh. Both are intensively used for agriculture, and natural habitat types have suffered significantly in the last millennia.

Comparing the extinct taxa with the threatened ones (CR + EN + VU), some key differences emerge. Considering critically endangered, endangered and vulnerable plants, the larger subregions such as West Pamirian B, South Tajikistanian A and Kuraminian reveal the highest changes; these regions have a great number of habitat types, high diversity of plant species and are still considerably changed by human population. Human activity in these areas consists not only of agriculture but also mining and grazing. An extensive part of the land – particularly in the South Tajikistanian subregion – is urbanised. In the cases of the most natural regions, such as the East Pamirian and Alaian, the high proportion of threatened plants is influenced by the considerable uniqueness of its flora and the great number of species that occur exclusively in these regions (15.3% and 8.8% of unique species in the subregion, occupying respectively the first and third position in the country; Fig. S7). The same pattern is observed in the South Tajikistanian C and A, Kuraminian and Prisyrdarian regions, with 11.2%, 5.6%, 7.8% and 6.4% of unique species, respectively. With regard to the EOO criterion, they gain a higher risk of extinction in the country (Fig. 2b).

Traditionally, mountain agro-pastoralism in Central Asia has been based on altitudinal transhumance, connected with livestock seasonal mobility up and down the slopes that allows for regrowth of pasture plants and is important for vegetation conservation^50,51^. Nowadays, privatisation of livestock is leading to changes in herding patterns; due to economic problems (including the high cost of transport and the poor condition of the roads), livestock are no longer moved to the more distant and highest pastures for summer grazing and stay longer in the valleys and plains^50,51^. It is possible that these changes have influenced the relationship observed by us, i.e. that the highest number of threatened species (CR + EN) is associated with lowlands and colline zones (Fig. 3).

The influence of the density of settlements is also apparent when we observe the elevation pattern of the threatened plants’ share. The lowlands, valley bottoms and winter pastures are the most impacted in terms of flora withdrawal (Fig. 3). For critically endangered and endangered taxa, the threshold altitude at which the share is higher than average is at ca. 3,000–3,200 m a.s.l., and for vulnerable taxa not much higher. This reflects the distribution of human population and, to some extent, the transformations of the habitats (Fig. S6).

When looking at the habitat requirements of extinct taxa, it is clear that the forest and shrub species are the most impacted (Fig. 4a), particularly those located in the upper montane belt just below the tree line. This can be related to overgrazing of forests and intensive, however irregular, forestry. A decrease in fuel and coal supply resulted in their price rise, hence the people are forced to use slow-growing shrubs for heating and cooking^51^. Around 50% of the forests have disappeared in the past 100 years in Tajikistan, causing massive soil erosion and increased risk of landslides. Several types of riverside forests, e.g. stands of *Fraxinus sogdiana*, *Populus pruinosa*, *Platanus orientalis*, have almost entirely vanished. This, in combination with soil erosion and overgrazing, strongly hampers the reestablishment of tree stands. Grazing often leads to denudation and soil degradation and consequently to desertification^53^, which almost excludes the potential recovery of natural forest vegetation and fails to sustain the populations of forest taxa. The second group most affected is grassland flora along all the altitudinal belts (from colline dry meadows up to the alpine swards and forbs). In this case, the main factor responsible is connected with overgrazing.

### Climate change vulnerability

The mountains of the Pamir-Alai are particularly sensitive to climate change due to the low adaptive capacity of its ecosystems^29^. Projections suggest a significant increase in temperature along with rainfall decline during the spring, summer and autumn seasons^54^. This, in combination with the drop of shepherding efficiency, may lead to further degradation of the vegetation cover. Moreover, desertification causes considerable threats to Tajik flora^37^ as the average temperature in the southern regions of the country rises by ca. 1 °C. Climate change in Central Asia is already noticeable via glacier melting in the higher elevations. During the twentieth century, the glacier area of the Tian Shan and Pamir-Alai decreased by 25–35%, which clearly indicates warming^55,56^.

Considering the threatened species (CR + EN + VU), the most endangered group is related to extreme habitats such as water bodies, deserts and semi-deserts (Fig. 4b). It is commonly known that these types of habitats are fragile to climate change^57,58^. In fact the authors observed a serious decline of shallow saline lakes in the upper altitudes and strong pollution in lower rivers and water bodies in the lowlands. In addition, the share of threatened species in the phytogeographical groups to some extent highlights the effect of climate change, as the highest proportion of threatened taxa has its origin in Central Asia – a much colder and more temperate region than the Irano-Turanian or Mediterranean (Fig. S4). Plants adapted to colder environments reveal a stronger withdrawal tendency than those from warmer climates (e.g. Saharo-Sindian). Interestingly, the pluriregional and cosmopolitan taxa take advantage in the group of extinct taxa when its share is considered – examples are *Crassula aquatica* or *Poa infirma* that have a wide range, but in Tajikistan occur in single locations. However, as the number of species is accounted for, the most numerous group is clearly the Irano-Turanian that reflects the rarity of the number of taxa that are endemic to Tajikistan and harbored by distinct and threatened habitats^41^.

### More attractive plants and spring bloomers with a shorter flowering period face a higher risk of extinction

As was expected, species with alluring, beautiful flowers are at higher risk of extinction. The Tajik tradition is to harvest beautiful flowers from the wild and sell them along roads or at markets, and also cultivate plants in their home gardens. This love for nature and beauty unfortunately poses a higher threat to the local flora. The peak of the bulb trade is still a long way behind (e.g. tulips), however there is still an intensive harvest from the wild or simple smuggling of bulb plants, putting many of them at a high risk of extinction (e.g. *Eremurus albertii*, *E. korovinii*, *E. pubescens*, *Fritillaria eduardii*). These kind of threats are known as the most important risk factors for ornamental plants that are contributing to their decline^59,60^.

The early blooming plants in Tajikistan are relatively more threatened than the other groups of taxa. This pattern is not so evident, however considering the extinct and vulnerable taxa we can find the shift towards the beginning of the vegetation season. There are few available data on the extinction risk relation to the flowering functional traits based in the whole flora analysis. In the US, a comparison of common and rare taxa showed that an earlier and longer blooming was typical for common taxa^61^. The same result was found for Finland’s red-listed taxa – with the late bloomers more threatened^62^. This is contrary to our findings in respect of blooming period, however fully in accordance when considering flower duration. Probably the differences are due to the considerably distinct traditional harvest of early spring flowers in Tajikistan, if compared to the controlled situation – regulated by strict law – in the US and Finland. It can be also related to very variable altitudinal gradient across Tajikistan, whose total denivelation reaches more than 7,000 m a.s.l. Such a topography strongly influences the seasonal variation of the vegetation. The long-lasting spring and related flower harvest begins in February in south-western parts of the country and ends in early July in eastern plateaus of the high Pamir Mountains, impacting a lot of species across an extensive area. An explanation of a lower threat level of long flowering plants still requires more detailed study and would probably have to involve a range of correlated factors such as human impact on short blooming geophytes, plant-pollinator interactions and investment in sexual reproduction^63^.

### Useful plants are less threatened than those neglected in the human economy

Local people in Tajikistan commonly harvest a great number of native plants for different purposes. Many people rely on traditional medicinal plants to prevent and cure health disorders^64^. Despite the fact that harvesting of useful plants can cause a considerable threat to the flora^65^, in Tajikistan the usage of medicinal and food plants still seems to be traditional and sustainable, without having a strong influence on the local flora. Probably it is also due to the harsh mountainous relief of the country, with many unavailable and inaccessible lands where no harvest is possible. Another reason that the species used in traditional medicine and the local cuisine are less threatened may be the overrunning effect of other threats (land development, urbanisation, climate change), if compared to other regions. However, the rapid changes in the Tajik economy, infrastructure and population may have deteriorating effects on that sustainable balance.

### The need for improving plant diversity conservation in Tajikistan

In the Pamir-Alai, large protected areas have been established in recent decades that cover ca. 22% of the country’s territory^37^; however, only a few are devoted particularly to floristic diversity conservation. Additionally, a number of programmes and strategies have been developed to enhance biodiversity conservation and management of protected areas, although in practice they are insufficiently managed because governmental institutions are not able to monitor and manage all the valuable plant populations and vegetation types. The compilation of a comprehensive endangered species list will hopefully improve this situation. Raising the effectiveness of conservation in Pamir-Alai needs urgent action plans with the establishment of specific priorities for the hotspots of plant diversity. The identification of micro-hotspots (currently under preparation by the authors) within the global hotspot of the Mountains of Central Asia is necessary, particularly for the most threatened ecosystems such as forests and grasslands.

The need for a stronger national administration must also be emphasised to deal with biodiversity conservation, which must involve the provision of financial support by international organisations. At a national level, the system of nature conservation should be improved so as to take better account of centres of endemism, improving the connectivity of the ecological network and enhancing the adaptive capacity of the most sensitive areas by ensuring a balance between traditional management practices and the economic growth of local economies. All these aforementioned points would not be achievable without a thorough inventory of current species distribution, analyses of their population sizes and phenology changes. This knowledge is an indispensable but still neglected step in conservation actions in Tajikistan.

## Supplementary information

Supplementary information.

Supplementary information 2.

## Data Availability

All data generated or analysed during this study are included in this published article (and its Supplementary Information Files).
