# Supplementary material for: Red List of vascular plants of Tajikistan – the core area of the Mountains of Central Asia global biodiversity hotspot
Source: Sci Rep. 2020 Apr 10;10:6235. doi: 10.1038/s41598-020-63333-9 (PMC7148292; doi:10.1038/s41598-020-63333-9)
Supplement: Supplementary file 1 — Supplementary information. [file 41598_2020_63333_MOESM1_ESM.pdf]

Supplementary material

Red List of vascular plants of Tajikistan - the core area of the Mountains of Central Asia global biodiversity hotspot

Arkadiusz Nowak[1,2], Sebastian Świerszcz[2*], Sylwia Nowak[1], Hikmat Hisorev[3], Ewelina Klichowska[2,4], Anna Wróbel[4], Agnieszka Nobis[4] & Marcin Nobis[4,5*]

[1]Institute of Biology, University of Opole, Opole, 45-052, Poland

[2]Botanical Garden, Center for Biological Diversity Conservation, Polish Academy of Sciences, Warszawa, 02-976, Poland

[3]Institute of Botany, Plant Physiology and Genetics, Tajik Academy of Sciences, Dushanbe, 734017, Tajikistan

[4]Department of Taxonomy, Phytogeography and Palaeobotany, Institute of Botany, Jagiellonian University, Kraków, 30-387, Poland

[5]Research laboratory 'Herbarium', National Research Tomsk State University, Tomsk, 634050, Russia

*corresponding authors:

Sebastian Świerszcz, email: s.swierszcz@obpan.pl;

Marcin Nobis, email: m.nobis@uj.edu.pl

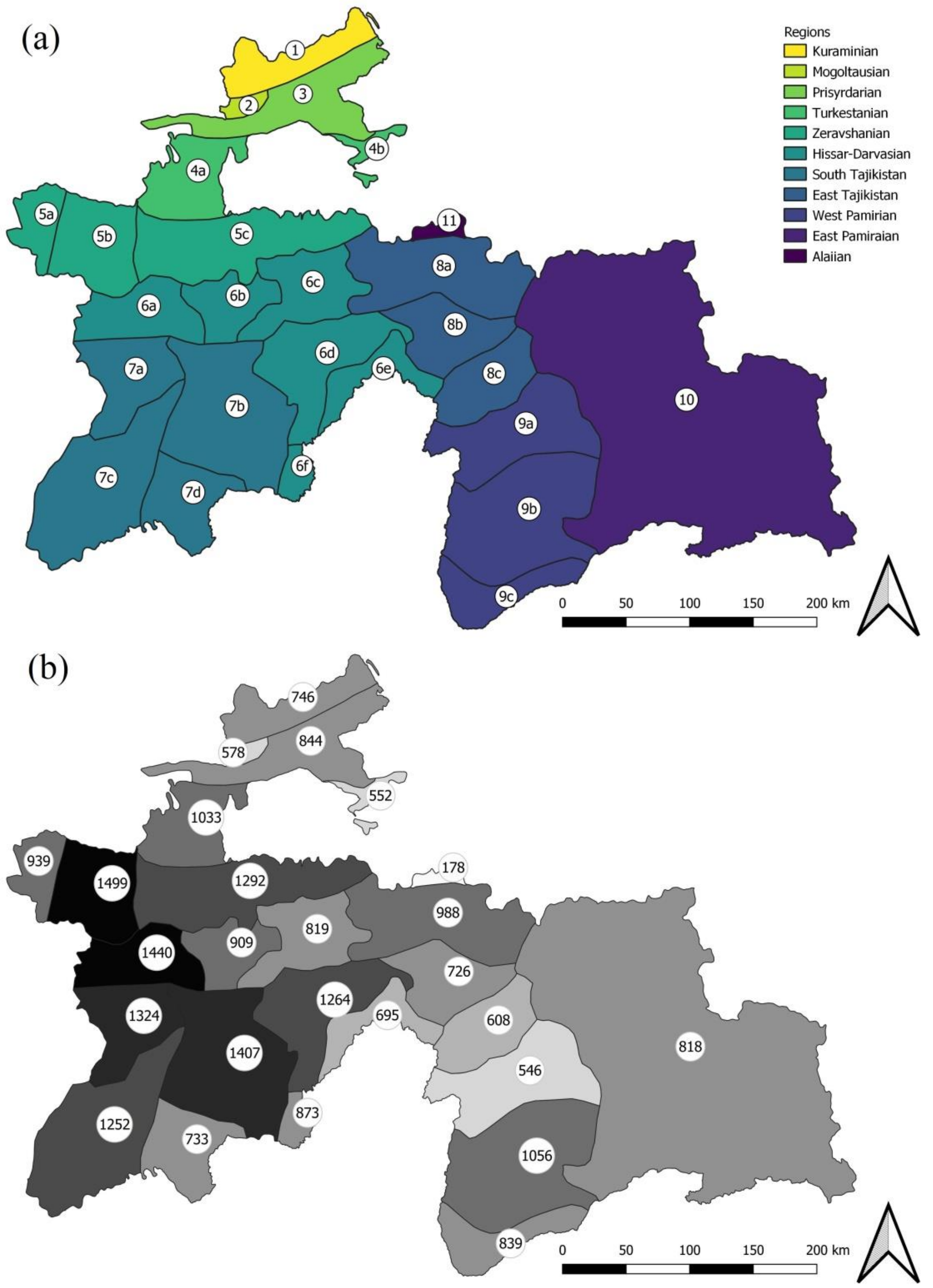

Figure S1. (a) The phytogeographical division of Tajikistan (subregions) and (b) floristic richness of subregions in Tajikistan – number of vascular plants in each subregion. Names of subregions in panel (a): 1 – Kuraminian, 2 – Mogoltausian, 3 – Prisyrdarian, 4a – Turkestanian A, 4b – Turkestanian B, 5a – Zeravshanian A, 5b – Zeravshanian B, 5c – Zeravshanian C, 6a – Hissar-Darvasian A, 6b – Hissar-Darvasian B, 6c – Hissar-Darvasian C, 6d – Hissar-Darvasian D, 6e – Hissar-Darvasian E, 6f – Hissar-Darvasian F, 7a – South Tajikistan A, 7b – South Tajikistan B, 7c – South Tajikistan C, 7d –South Tajikistan D, 8a – East Tajikistan A, 8b – East Tajikistan B, 8c – East Tajikistan C, 9a – West Pamirian A, 9b – West Pamirian B, 9c –West Pamirian C, 10 – East Pamiraian, 11 – Alaiian.

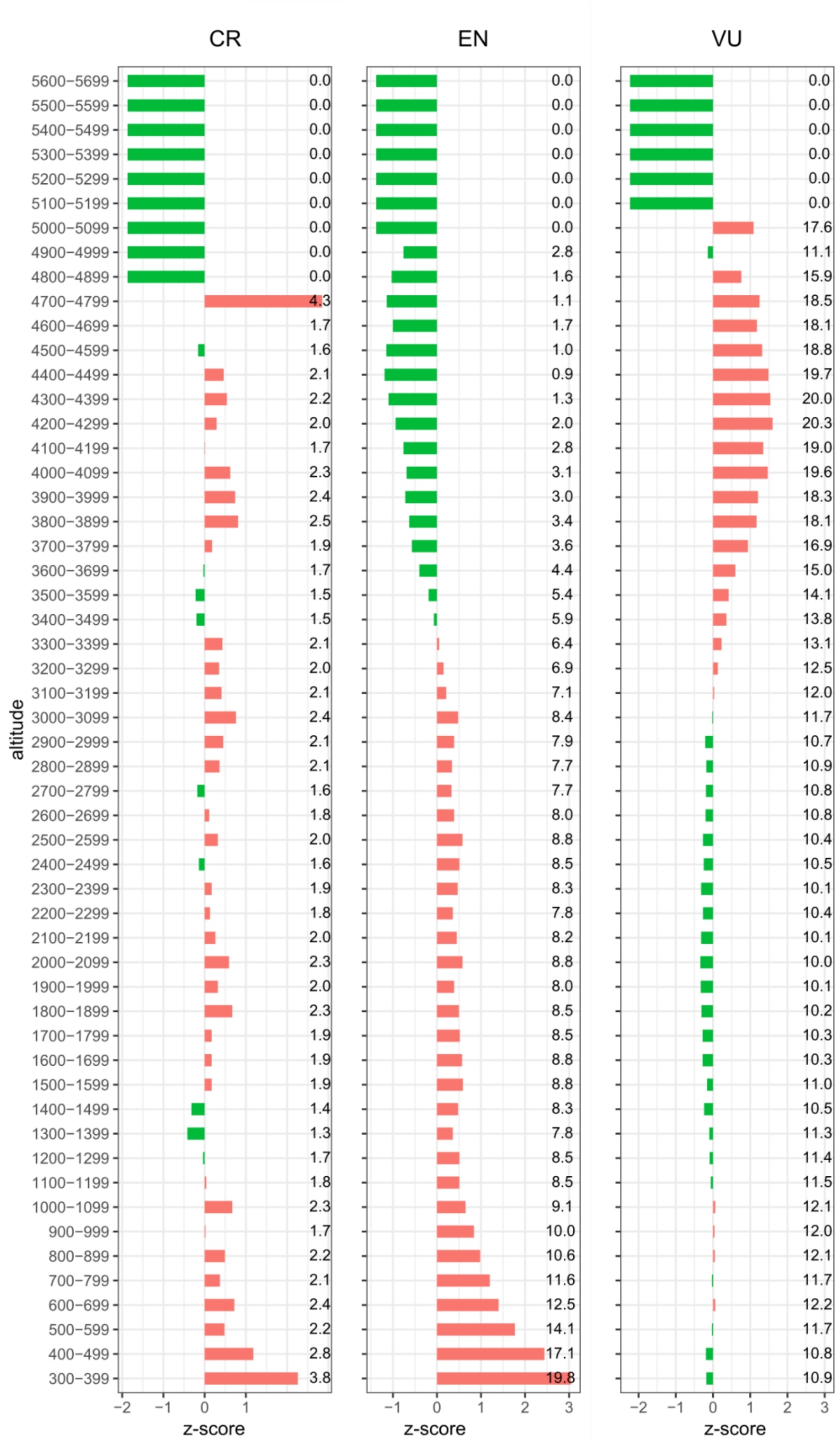

Figure S2. Deviations from the average number of species within altitudinal belts for the threatened categories CR, EN and VU expressed as z-scores. The numbers on the right side of the graphs presents the proportion of endangered species at a given altitudinal belt. Values above the average are shown in red and below average in green.

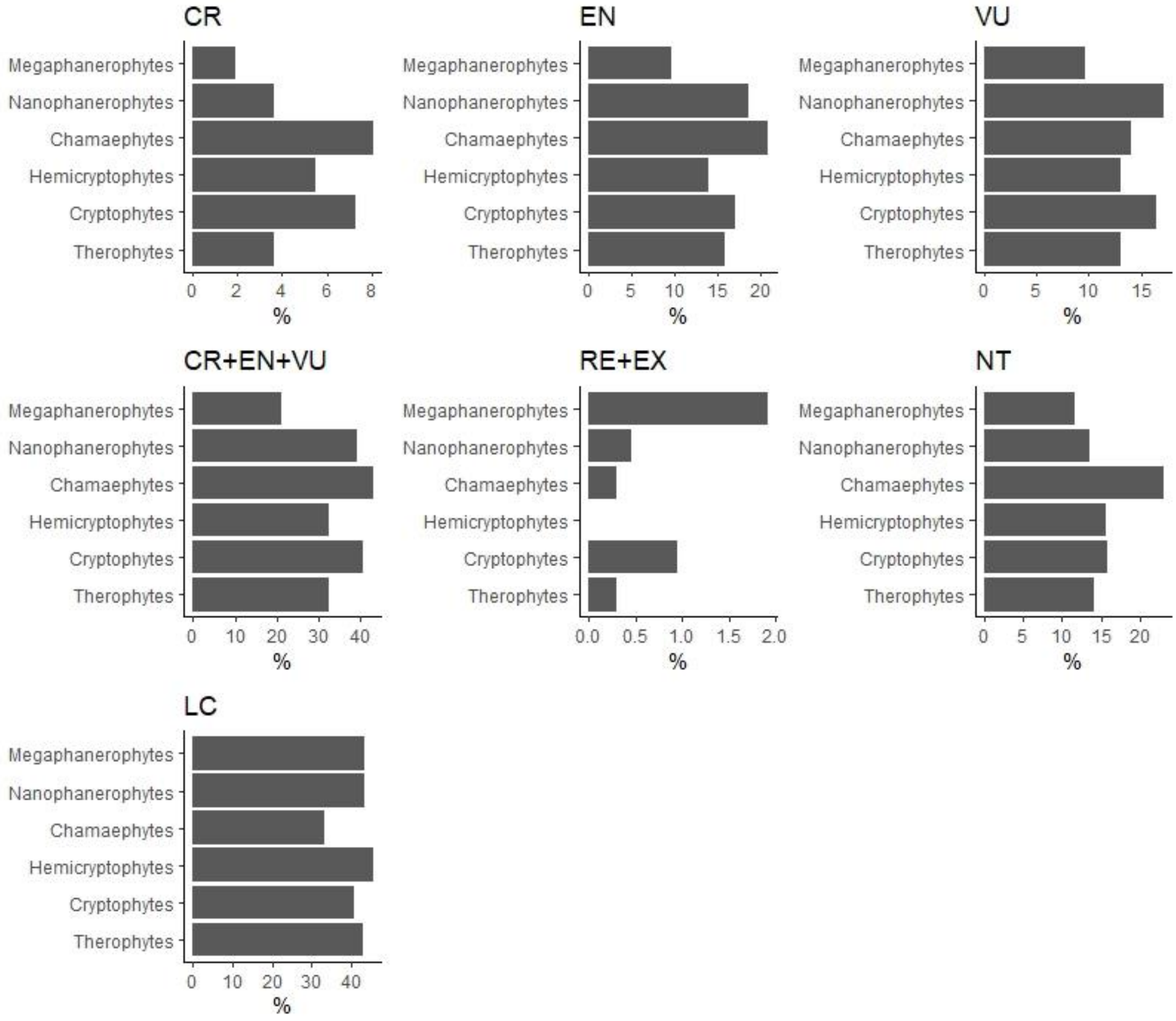

Figure S3. The proportion of species with different threat category with regard to their life form.

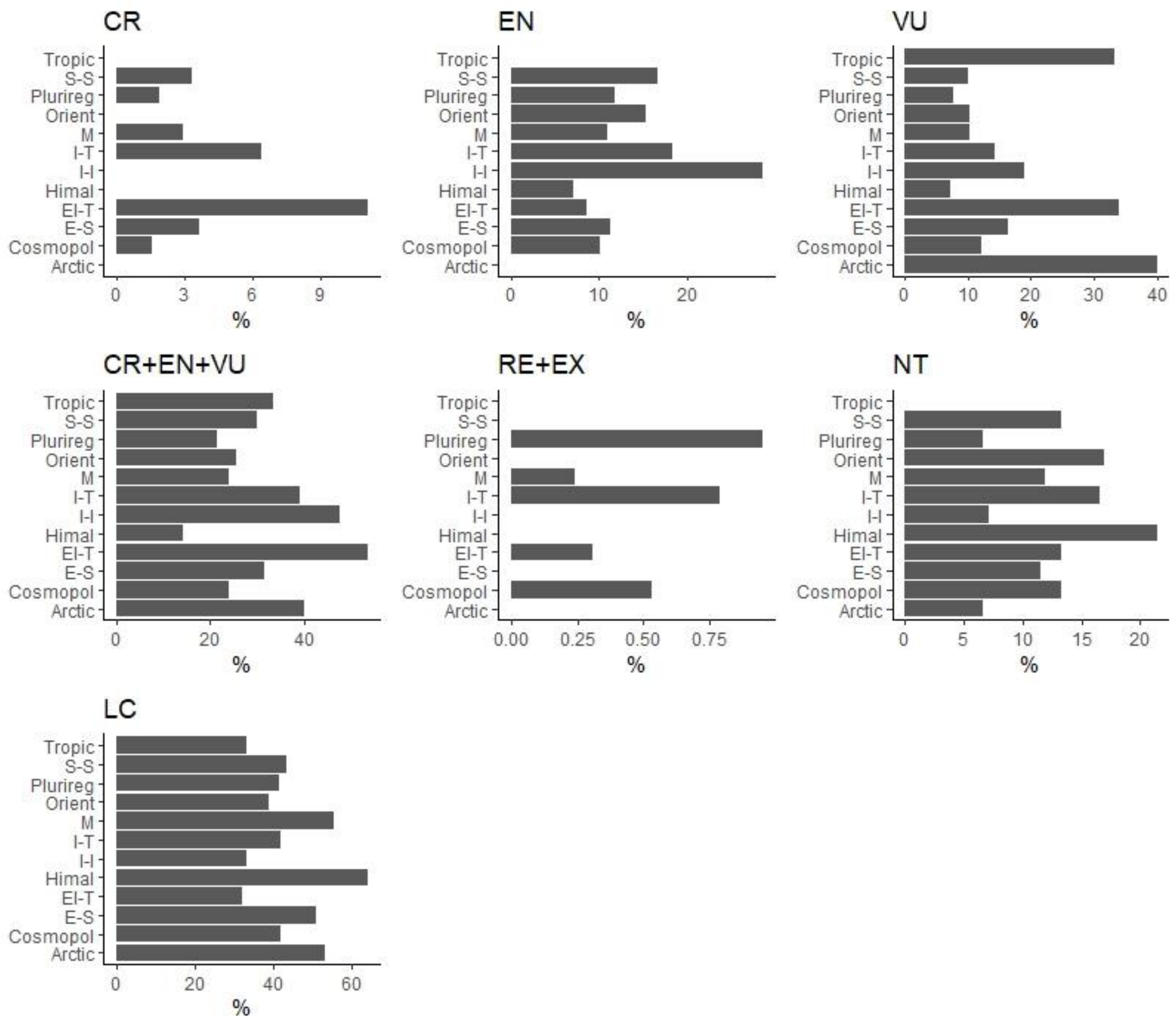

Figure S4. The proportions of species with different threat categories with regard to its geographical distribution. Abbreviations: Tropic – Tropical, S-S – Saharo-Sindian, Plurireg – pluriregional, Orient – Oriental, M – Mediterranean, I-T – Irano-Turanian, I-I – Indo-Indochinese, Himal – Himalayan, EI-T – Central Asiatic-Irano-Turanian, E-S – Euro-Siberian, Cosmopol – cosmopolitan.

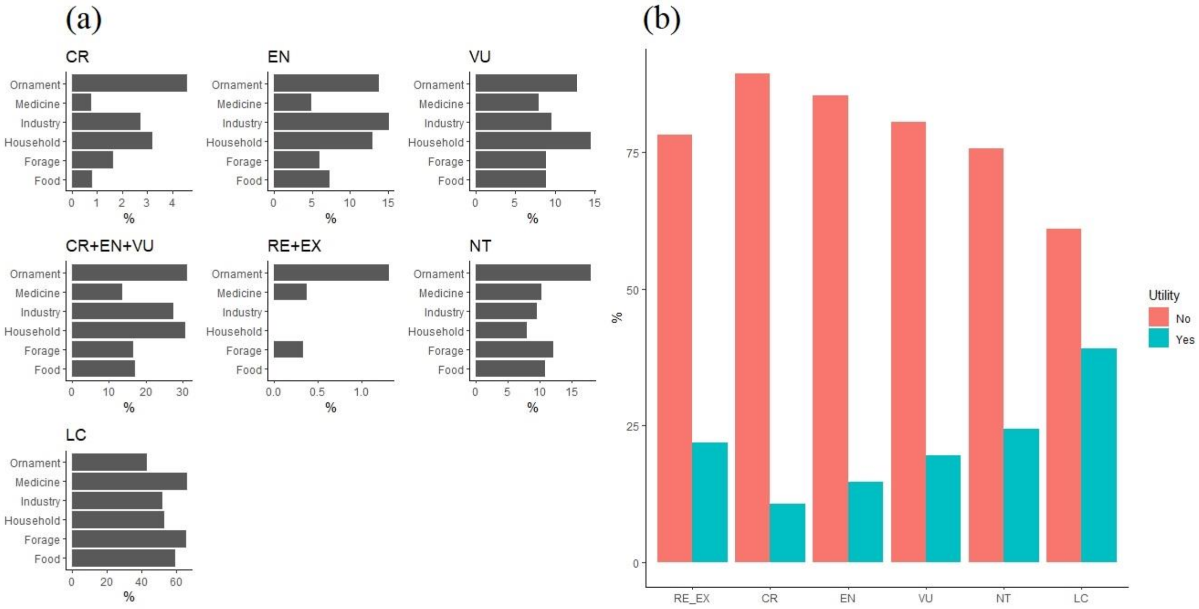

Figure S5. (a) The proportion of species with different threat categories with regard to its usage and (b) comparison of the usage types of species with different threat categories.

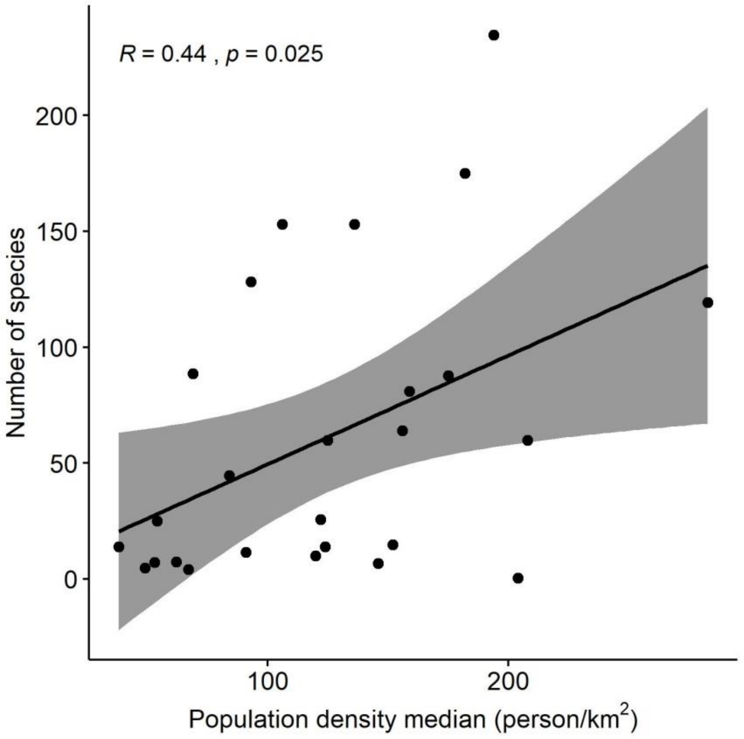

Figure S6. The correlation between the number of species with the threat category: CR, EN, VU, RE, EX and the median population density of each phytogeographical subregion in Tajikistan

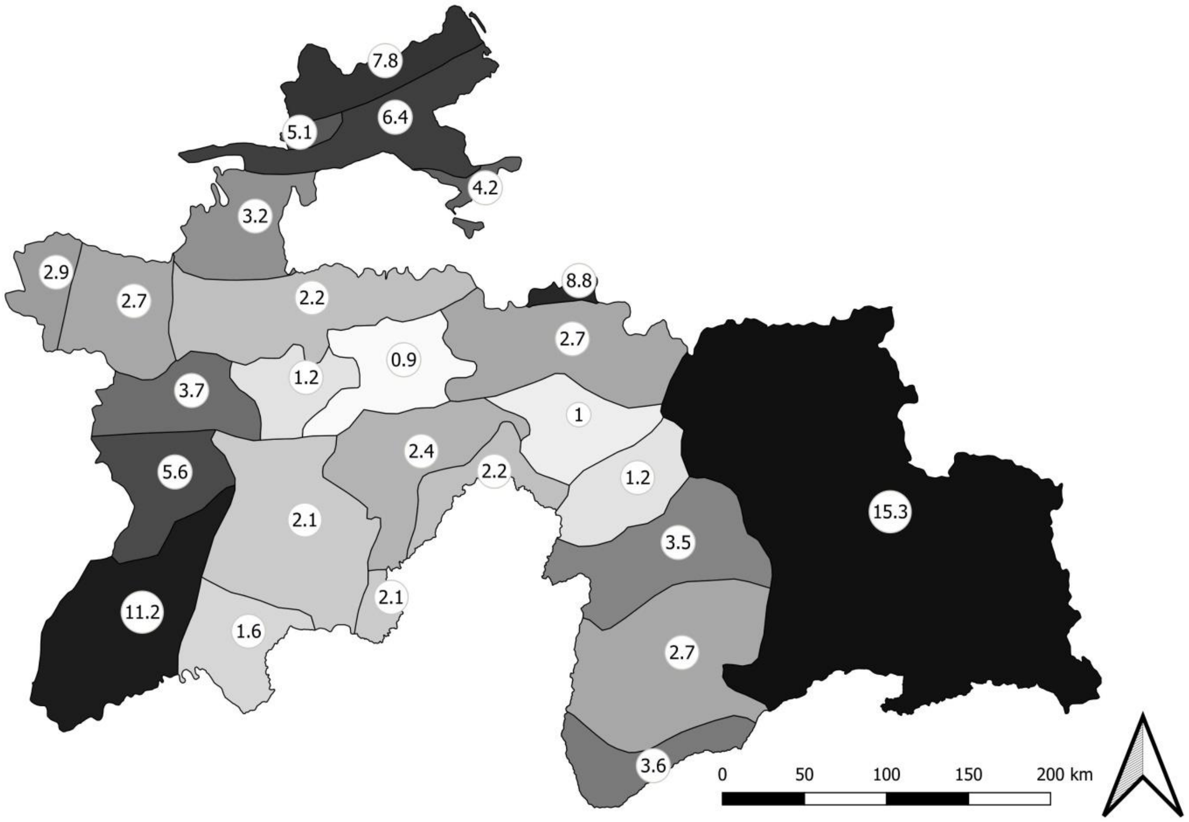

Figure S7. Percentage of unique species in 26 phytogeographical subregions.
